# P-2185. Management of CMV Prophylaxis in Lung Transplant Patients at a Large Academic Medical Center

**DOI:** 10.1093/ofid/ofaf695.2348

**Published:** 2026-01-11

**Authors:** Kaitlyn Reasoner, Andrea Ito, Richard M Merkhofer, Michael Zou, Kyle T Enriquez, Emily Moore, Barbara L Mora, Wonbeom Paik, Luke Pryke, Megan Uehling, Ashley Zeoli, Milner Staub, Augusto Dulanto Chiang, Casey Smiley

**Affiliations:** Vanderbilt University Medical Center, Nashville, TN; Vanderbilt University, Nashville, Tennessee; Vanderbilt University Medical Center, Nashville, TN; Vanderbilt University, Nashville, Tennessee; Vanderbilt University Medical Center, Nashville, TN; Vanderbilt University Medical Center, Nashville, TN; Vanderbilt University Medical Center, Nashville, TN; Vanderbilt University Medical Center, Nashville, TN; Vanderbilt University Medical Center, Nashville, TN; Vanderbilt University Medical Center, Nashville, TN; Vanderbilt University Medical Center, Nashville, TN; Vanderbilt University Medical Center, VA Tennessee Valley Healthcare System, Nashville, TN; Vanderbilt University Medical Center, Nashville, TN; Vanderbilt University Medical Center, Nashville, TN

## Abstract

**Background:**

Standard cytomegalovirus (CMV) prevention in transplant recipients uses antiviral prophylaxis based on risk stratification by CMV serology. The American Society of Transplantation updated guidelines for CMV management in 2025, but practice varies widely. Lung transplant prophylaxis durations at Vanderbilt University Medical Center (VUMC) tend to be shorter than other institutions with maximum duration of 9 months for high-risk serotypes (Fig 1). This project aimed to assess current practice variation and assess organization readiness for a standardized approach incorporating CMV T-cell immunity testing at Vanderbilt University Medical Center (VUMC).

**Table 1:**
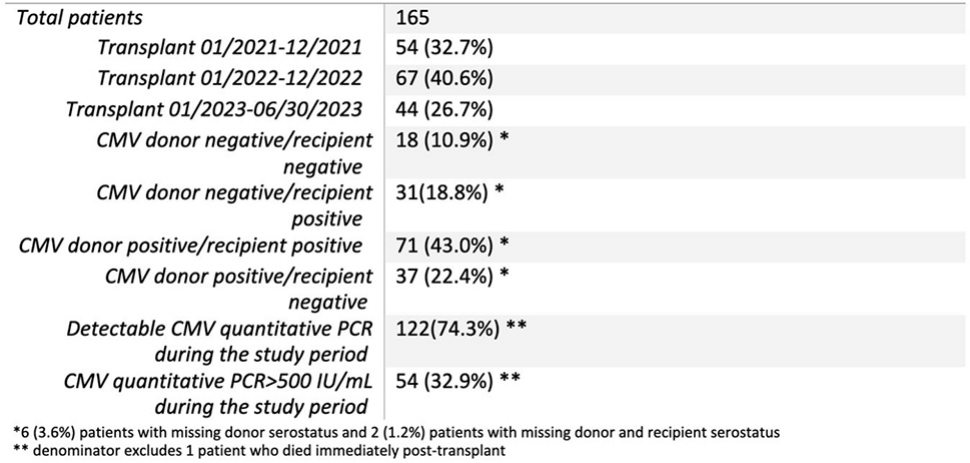
Study population of lung transplant recipients at VUMC between January 2021 and June 2023.

**Figure 1:**
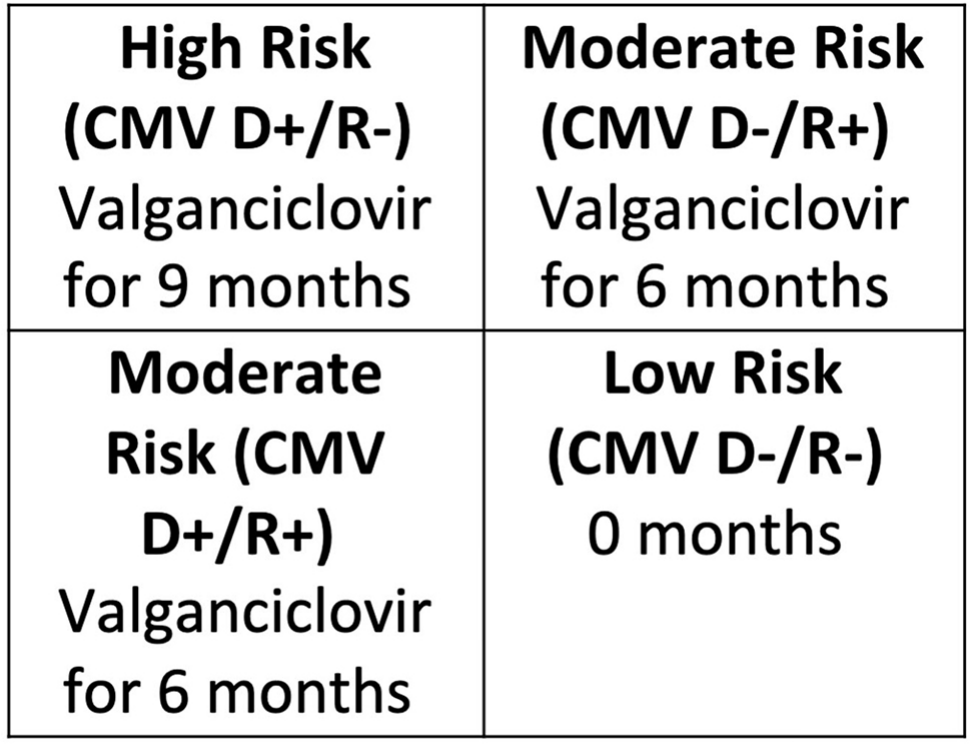
Current primary CMV prophylaxis protocol for lung transplants at VUMC.

**Methods:**

To quantify the need for updated CMV prophylaxis protocols, we evaluated the total number and positive proportion of CMV tests in the 18-month post-transplant period for VUMC patients who received a lung transplant from 1/2021-6/2023. A pre-implementation organizational readiness for change (ORC) survey was administered to all VUMC lung transplant clinic providers evaluating CMV prophylaxis prescribing practices, perceived adequacy of the current process, and perceptions of T-cell mediated immunity tests for CMV risk stratification.

**Figure 2.**
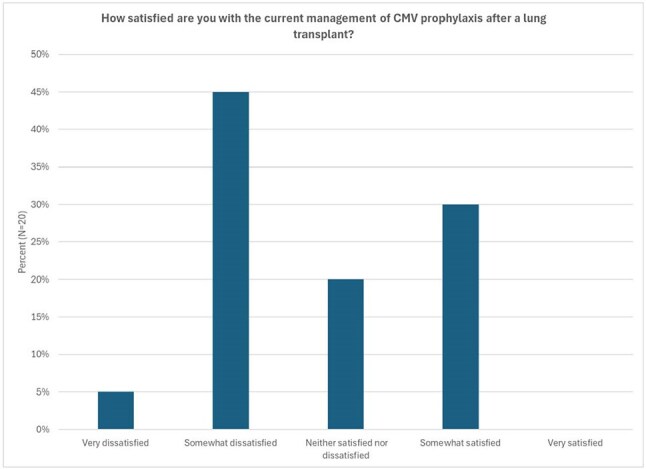
Survey of lung transplant clinic providers (N=20) regarding satisfaction with current CMV prophylaxis management.

**Figure 3.**
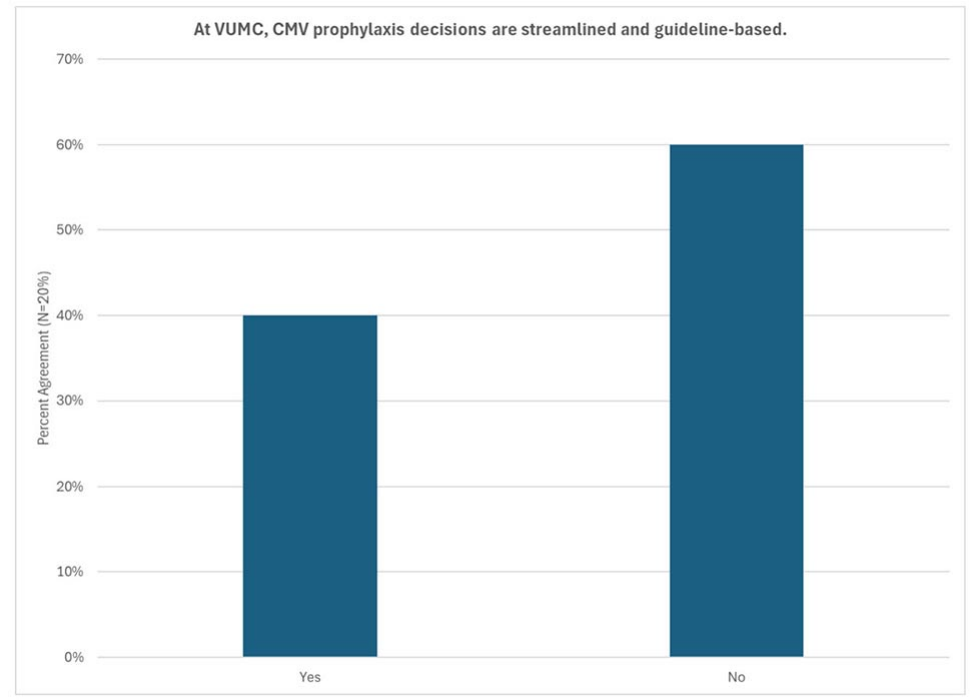
Survey of lung transplant clinic providers (N=20) regarding perception of CMV prophylaxis decisions.

**Results:**

During the study period, 165 patients collectively had 4485 CMV quantitative PCR tests, and 54 patients (32.9%) had a CMV quantitative PCR >500 IU/mL (Table 1). Twenty lung transplant clinic providers completed ORC surveys (Figures 2 & 3), and 7 (46.7%) providers felt patients received appropriate CMV prophylaxis durations, but 15 (66.7%) providers felt prophylaxis duration was changed somewhat or very often due to valganciclovir toxicities or cost.

**Conclusion:**

Our results suggest there are opportunities for improving the VUMC CMV prophylaxis process for lung transplant patients and clinic providers. VUMC plans to implement a T-cell-mediated immunity test which may improve CMV risk-stratification and optimize antiviral prophylaxis duration, thereby reducing direct and indirect effects of CMV infection and antiviral toxicities. Forthcoming data on patient-specific factors that increase reactivation risk may aid a more nuanced risk stratification and testing strategy.

**Disclosures:**

Kaitlyn Reasoner, MD, Kindle Direct Publishing (Amazon): Royalties from a self-published book unrelated to topic of abstract Milner Staub, MD, MPH, Eli Lilly: Stocks/Bonds (Public Company)|Gilead: Stocks/Bonds (Public Company)|Johnson & Johnson: Stocks/Bonds (Public Company)

